# Dairy Consumption, Lactase Persistence, and Mortality Risk in a Cohort From Southern Sweden

**DOI:** 10.3389/fnut.2021.779034

**Published:** 2021-11-24

**Authors:** Emily Sonestedt, Yan Borné, Elisabet Wirfält, Ulrika Ericson

**Affiliations:** ^1^Nutritional Epidemiology, Department of Clinical Sciences Malmö, Lund University, Malmö, Sweden; ^2^Diabetes and Cardiovascular Disease–Genetic Epidemiology, Department of Clinical Sciences Malmö, Lund University, Malmö, Sweden

**Keywords:** dairy, milk, cheese, butter, mortality, LCT-13910C/T polymorphism, cohort, epidemiology

## Abstract

**Background:** Whether high dairy consumption is related to longevity is still unclear, and additional studies of prospective cohorts with high-quality dietary data from populations with wide consumption ranges are needed.

**Objective:** To examine the association between dairy consumption and mortality in a Swedish cohort.

**Design:** Among 26,190 participants (62% females, 45–73 years old) without diabetes and cardiovascular disease from the population-based Malmö Diet and Cancer cohort, 7,156 individuals died during a mean follow-up time of 19 years. Data on intake of dairy (non-fermented milk, fermented milk, cheese, cream and butter) were collected from 7 day food records and food questionnaires. A genetic marker (rs4988235) associated with lactase persistence was detected among 22,234 individuals born in Sweden.

**Results:** Higher intakes up to 1,000 g/day of non-fermented milk were associated with only marginal higher mortality rates after adjusting for potential confounders. However, intakes above 1,000 g/day (1.5% of the population) were associated with 34% (95% CI: 14, 59%, p-trend=0.002) higher mortality compared to that with < 200 g/day. Fermented milk and cheese intake were inversely associated with mortality. Cream showed a protective association only among men. Butter was not associated with mortality. CT/TT genotype carriers (i.e., individuals with lactase persistence) had a 27% higher reported consumption of non-fermented milk, and non-significant higher mortality risk (HR = 1.08; 95% CI = 0.96, 1.23; *p* = 0.20) than CC genotype carriers.

**Conclusions:** Higher mortality rates were mainly observed among participants consuming more than 1,000 g of non-fermented milk per day. In contrast, fermented milk and cheese were associated with lower mortality. Because dairy products differ in composition, it is important to examine them separately in their relation to health and disease. The use of a genetic variant as an objective marker of lactose-containing milk intake should be examined in relation to mortality in a larger population.

## Introduction

Whether high consumption of dairy products is related to longevity is still unclear (1–5). In observational studies, high consumption of dairy products has not consistently been related to total or disease-specific mortality (1–9). Because dairy products differ in their composition and processing, for example fat content and whether they are fermented, it is important to examine them separately in their relation to health and disease. Substantial heterogeneity driven by sex, country and study quality has been shown when examining the association between non-fermented milk consumption and mortality (1). Given the observed heterogeneity in results between studies, additional studies of prospective cohorts with high-quality dietary data from populations with wide consumption ranges of diverse dairy products are required. For example, studies examining the risk with very high intake levels (i.e., more than 1 liter of milk per day) are lacking. Comprehensive and detailed data on dairy food consumption are advantageous when examining the association between diet and disease risk.

In addition, observational studies can be prone to confounding and recall bias. One way to overcome this problem is to use a genetic marker as a proxy for long-term intake. Genetic variants are assigned randomly at conception and are not predisposed to confounding by lifestyle factors. The rs4988235 single nucleotide polymorphism (SNP, *LCT*-13910) located upstream of the lactase persistence gene (*LCT*) has consistently been associated with lactase persistence (i.e., lactose tolerance) among adults of European ancestry (10). Lactase persistence is inherited as a dominant trait, and individuals carrying the T-allele continue express the lactase gene after childhood. On the other hand, individuals homozygous for the C-allele exhibit lower lactase enzyme activity in adulthood and may avoid lactose-containing dairy products (mainly non-fermented milk) because of gastrointestinal symptoms (11).

The aim of this project was to examine the association between different types of dairy products (non-fermented milk, fermented milk, cheese, cream and butter) and the rs4988235 SNP with all-cause mortality using data of the Malmö Diet and Cancer (MDC) cohort.

## Materials and Methods

### Subjects and Data Collection

The MDC study is a population-based cohort with baseline examinations conducted between 1991 and 1996. All women born between 1923 and 1950 and men born between 1923 and 1945 living in Malmö were invited to participate in the study (*n* = 74,318) (12). The participants had to visit the study center twice. During the first visit, questionnaires were handed out, anthropometrics were recorded, and non-fasting blood were collected. During the second visit, the questionnaires were checked for completeness and a 1 h diet interview was conducted. In total, 30,446 individuals took part in the baseline examination, and complete diet, questionnaire and anthropometric data were available for 28,098 (60% women) individuals. The Ethics Committee at Lund University approved the study (nr 51–90). After excluding individuals with a history of myocardial infarction or stroke from national registers (*n* = 820), a self-reported diabetes diagnosis or the use of diabetes medication at baseline (*n* = 870), and with missing information on smoking (*n* = 12), body mass index (BMI) (*n* = 42), leisure-time physical activity (*n* = 191), or educational level (*n* = 71), 26,190 individuals remained and constituted the study sample.

### Diet Assessment Method

A modified diet history method specifically developed for the MDC cohort was used to estimate habitual dietary intake (13, 14). The method is a combination of three parts: a food record, a questionnaire and an interview. The following food groups were examined in this project: non-fermented milk, fermented milk (yogurt and sour milk), cheese (>10% fat), cream and butter (including the butter/vegetable oil-based spread “Bregott”) (15). Both dairy products used in cooking and milk consumed separately were included. In 7 day food records, participants had to record prepared meals (usually cooked lunches and dinners), the amount of intake of cold beverages and dietary supplements. In the food record, information was collected on the use of dairy products in cooked meals, and the amount of milk consumed (as a drink) from a list of four prespecified types of milk with various fat contents. In a 168-item diet questionnaire, questions were asked regarding the frequency and serving sizes over the past year of food items not covered in the food record (mainly breakfasts and snacks). In the diet questionnaire, questions were asked about milk and cream in coffee, milk in tea, chocolate milk, butter and milk-based spread on bread, cheese on bread, other cheese consumed (e.g., cheese plates), milk in porridge, milk on cereals, milk and cream on fruit compote and yogurt and other fermented milks (e.g., sour milk). During a 45–60 min diet history interview, questions were asked about food preparation methods and serving sizes for meals recorded in the food record. The average daily intake of foods (g per day) was calculated by summing the intakes from the diary and the questionnaire. In September 1994, a change in coding to reduce the interview time did not influence the ranking of the individuals according to dietary intake (14).

In a validation study, a slightly different diet assessment method (130-item questionnaire and 2 week food records) was compared against a reference method of 18-day weighted food records collected over 1 year among 206 individuals living in Malmö in 1984–85 (13, 16). The energy-adjusted correlation coefficients were as follows: for milk, 0.83 and 0.84 for men and women, respectively; for cream 0.47 and 0.52; and for cheese 0.47 and 0.59. When compared against data obtained with the reference method, 54% of the participants in the highest quartile for milk intake were correctly classified (13).

We categorized individuals according to predefined intake levels (i.e., for non-fermented milk: 0–200, 200–400, 400–600, 600–800, 800–1,000 and >1,000 g; fermented milk: 0, 0–100, 100–200, 200–300, >300 g; cheese: 0–20, 20–40, 40–60, 60–80, 80–100, >100 g; cream: 0–10, 10–20, 20–30, 30–40, 40–50, >50 g; and butter: 0, 0–10, 10–20, 20–30, 30–40, 40–50, >50 g). The intakes were also energy-adjusted using the residual method and standardized according to the median energy intake in this population (9.55 MJ). Participants were thereafter categorized based on their energy-adjusted intake values.

### Mortality

We followed the participants from study entry until death, emigration or end of follow-up on 31 December 2014. Information on vital status and emigration was obtained from the National Tax Board.

### Selection of the Genetic Variant and Genotyping Method

The SNP rs4988235 (LCT-13910 C/T), which was selected as a proxy for milk intake, was available from genome-wide screening. The Illumina GSA v1 genotyping array was used for genotyping. rs4988235 was not among the SNP that were genotyped directly, and we used imputed values using the Haplotype Reference Consortium reference panel (17). In our study sample, we had genotype information for 25,242 individuals (96%). After restricting our study sample to participants born in Sweden (*n* = 22,234), the SNP had a minor allele frequency of 0.21 and was in Hardy Weinberg Equilibrium (*p* = 0.53).

### Other Variables

Individuals were categorized into three groups according to their smoking habits: current smokers, never-smokers and former smokers. Individuals were categorized into five groups based on their educational status: elementary school, primary and secondary school, upper secondary school, further education without a degree, and University degree. The participants reported the duration they spent on 17 different leisure-time physical activities. The duration (hours per week) of each activity was multiplied by a metabolic equivalent of task (MET) factor based on intensity and summed to MET h per week of leisure-time physical activity. Individuals were categorized into six groups: 0–7.5, 7.5–15, 15–25, 25–50, and >50 MET h per week. Alcohol habits were estimated from the questionnaire and 7 day diaries. Individuals were categorized into six groups: zero-consumers (no alcohol consumption during the previous year and no reported consumption in the 7 day record) and sex-specific quintiles (based on the consumption reported in the 7 day record). BMI was calculated from measured weight and height. Individuals reporting a substantial diet change before baseline in the questionnaire were characterized as diet changers (*n* = 5,684) (18). Potential misreporters (*n* = 4,806) were defined as having a reported energy intake to basal metabolic rate ratio outside of the 95% confidence interval (CI) of the physical activity level (estimated from information on physical activity at work, during leisure time, household work, estimated sleeping hours, self-care, and passive time (19).

### Statistical Analyses

Cox proportional hazards regression was used to calculate hazard ratios (HRs) and 95% CIs for different intakes of dairy products (both absolute and energy-adjusted intakes) associated with all-cause, with time of follow-up as the underlying time variable. The potential confounders were selected *a priori* based on the literature. The proportionality hazard assumption was tested with Shoenfeld statistics. The proportions assumption was slightly improved when the model was stratified for smoking and alcohol habits. However, using smoking and alcohol habits as stratified variables only marginally influenced the HR estimates and was therefore not used in the models presented. In the basic model, we adjusted for age (continuous) and sex. In the full model, we adjusted for season (spring, summer, autumn, or winter), diet assessment method (before or after 1 September 1994 when the interview time was reduced from 60 to 45 min), energy intake (continuous), BMI (continuous), smoking habits (three categories), alcohol habits (six categories), educational status (five categories), leisure-time physical activity (five categories), and intake of fruit and vegetables (continuous), meat (continuous), fiber (continuous), and sugar-sweetened beverages (continuous). In addition, we tested for interactions between sex and the food variables by introducing a multiplicative factor into the model. In sensitivity analyses, we excluded individuals reporting past food habit changes and those identified as potential misreporters of energy intake (35 % of the population). To examine the shape of the association, a restricted cubic spline regression with 4 knots was computed for men and women separately using the model with full adjustments. The analyses with the lactase persistence rs4988235 variant were restricted to participants born in Sweden. Differences in dietary intakes between genotypes were computed with a general linear model adjusted for age, sex, energy intake, season, and diet assessment method. Cox regression was used to examine the association between the variant and mortality using the additive and dominant model adjusted for age and sex. Mendelian Randomization analysis was performed using a two-stage least squares analyses with rs4988235 as instrumental variable in a logistic regression model. SPSS version 26 (IBM Corporation, Armonk, NY, US) and Stata 17 (StataCorp, College Station, TX, US) were used for the statistical analyses.

## Results

The average reported intakes were: 311 g/day for men and 256 g/day for women of non-fermented milk, 81 g/day for men and 91 g/day for women of fermented milk, 44 g/day for men and 41 g/day for women of cheese, 16 g/day for men and 14 g/day for women of cream, and 15 g/day for men and 9 g/day for women of butter. Participant characteristics across intake groups are shown in Table 1. For example, high intake of non-fermented milk was associated with higher BMI, lower number of women, lower educational level, and lower alcohol consumption. High intakes of fermented milk and cheese were associated with a lower age and higher educational level.

**Table 1:** Participant characteristics according to intake groups of dairy products.

**Non-fermented milk**							
**Intake groups (g/day)**	0–200	200–400	400–600	600–800	800–1000	>1000	
*N*	11,655	8,011	4,155	1,482	495	392	
Age, y	57.2 (7.5)	58.4 (7.7)	58.6 (7.7)	58.3 (7.6)	57.6 (7.6)	57.1 (6.8)	
BMI, kg/m^2^	25.3 (3.8)	25.7 (3.9)	25.9 (4.0)	26.1 (4.2)	26.4 (4.3)	26.5 (4.2)	
Smokers (%)	26.4	27.7	29.8	34.0	35.2	51.8	
Women (%)	64.5	64.6	60.0	50.6	43.8	24.5	
University degree (%)	16.3	13.9	12.3	11.7	14.3	11.0	
Zero-consumers of alcohol (%)	4.2	6.0	8.9	9.0	11.9	12.5	
Low leisure-time physical activity (%)	9.3	8.8	9.7	10.1	11.5	13.8	
**Fermented milk**							
Intake groups (g/day)	0	0–100	100–200	200–300	>300		
*N*	9,102	7,940	5,728	2,364	1,056		
Age, y	58.4 (7.5)	57.3 (7.7)	57.9 (7.6)	57.9 (7.5)	57.3 (7.5)		
BMI, kg/m^2^	25.8 (4.0)	25.6 (4.0)	25.6 (3.9)	25.2 (3.5)	25.2 (3.6)		
Women (%)	51.5	70.1	68.1	63.2	56.3		
Smokers (%)	33.4	27.4	24.1	24.1	24.4		
University degree (%)	10.3	14.7	16.9	20.3	24.3		
Zero-consumers of alcohol (%)	7.3	5.5	5.0	5.3	7.0		
Low leisure-time physical activity (%)	12.2	8.5	7.3	7.1	7.9		
**Cheese**							
Intake groups (g/day)	0–20	20–40	40–60	60–80	80–100	>100	
*N*	6,211	8,470	5,885	2,919	1,469	1,236	
Age, y	59.5 (7.6)	58.4 (7.7)	57.2 (7.4)	56.3 (7.2)	55.8 (7.2)	55.5 (6.9)	
BMI, kg/m^2^	26.0 (4.0)	25.7 (4.0)	25.5 (3.9)	25.4 (3.8)	25.2 (3.9)	25.2 (4.1)	
Women (%)	63.2	63.8	62.1	60.0	56.4	54.6	
Smokers (%)	28.8	27.2	27.7	29.3	29.7	32.5	
University degree (%)	9.3	13.0	16.4	18.5	22.2	24.4	
Zero-consumers of alcohol (%)	9.8	5.2	4.9	4.8	3.6	4.4	
Low leisure-time physical activity (%)	11.0	9.2	8.7	8.0	9.1	9.1	
**Creme**							
Intake groups (g/day)	0–10	10–20	20–30	30–40	40–50	>50	
*N*	12,483	6,929	3,405	1,624	831	918	
Age, y	57.5 (7.5)	57.8 (7.7)	58.4 (7.9)	58.7 (7.5)	58.9 (7.5)	59.3 (7.1)	
BMI, kg/m^2^	25.9 (4.1)	25.5 (3.8)	25.4 (3.8)	25.2 (3.5)	25.3 (3.8)	25.0 (3.6)	
Women (%)	62.6	64.3	62.4	58.6	52.9	50.5	
Smokers (%)	30.2	25.9	26.7	27.0	28.3	30.0	
University degree (%)	13.8	15.4	15.7	15.5	13.5	12.9	
Zero-consumers of alcohol (%)	7.6	4.6	4.4	4.8	4.9	5.0	
Low leisure-time physical activity (%)	10.6	8.3	8.3	8.1	7.1	8.5	
**Butter**							
Intake groups (g/day)	0	0–10	10–20	20–30	30–40	40–50	>50
N	14,826	3,671	2,185	1,780	1,349	768	1,611
Age, y	58.1 (7.6)	57.1 (7.6)	57.2 (7.7)	57.6 (7.6)	58.1 (7.8)	58.5 (7.3)	58.4 (7.4)
BMI, kg/m^2^	25.9 (3.9)	25.6 (3.9)	25.3 (3.9)	25.2 (3.9)	25.1 (4.0)	24.7 (3.6)	24.9 (3.8)
Women (%)	62.2	71.4	66.5	65.7	58.7	50.8	36.9
Smokers (%)	25.5	24.7	30.1	33.8	36.0	38.3	43.3
University degree (%)	12.8	19.9	18.2	17.2	14.3	11.8	11.7
Zero-consumers of alcohol (%)	6.7	4.4	5.9	5.7	5.7	4.3	6.0
Low leisure-time physical activity (%)	9.2	8.0	10.6	9.6	11.1	10.8	10.2

*Low physical activity = < 7.5 MET-h/wk of leisure-time physical activity*.

**Figure 1:**
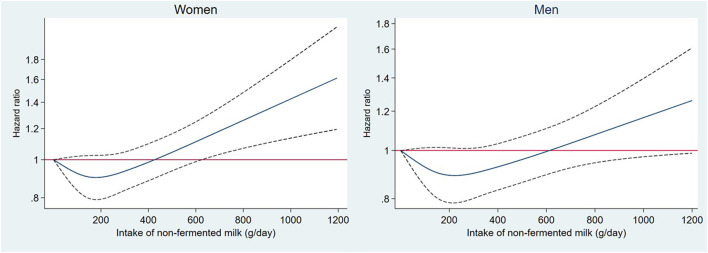
Association between non-fermented milk intake (grams per day) and morality rate modeled using a restricted cubic spine with four knots in 26,190 participants from the Malmö Diet and Cancer Study. The model was adjusted for adjusted for age, sex, method, season, energy, BMI, education, physical activity, smoking, alcohol habits, and diet (fruit and vegetables, meat, fiber, sugar-sweetened beverages). Solid line is Hazard Ratio and dotted line is 95% CI.

During a mean follow-up time of 19 years (range: 0–24 years) and 488,989 person years, 7,156 individuals (27%) died, and 245 individuals emigrated. In the basic model, high intake of non-fermented milk and butter were associated with higher all-cause mortality, while high intakes of fermented milk, cheese and cream were associated with lower mortality. After adjusting for several potential confounding factors, non-fermented milk, fermented milk and cheese remained statistically significantly associated with all-cause mortality. Using energy-adjusted values had only a minor influence on the HR estimates (Table 2). Spline regression was performed to evaluate the shape of the associations (Figure 1; Supplementary Figure 1). We observed a J-shaped association between non-fermented milk intake and mortality in both women and men, with the lowest mortality rate observed at approximately 200 g per day (Figure 1). Compared to the group of individuals consuming < 200 g per day of non-fermented milk (i.e., <one glass/day), intakes in the range of 200 to 1,000 g per day of non-fermented milk (i.e., two to five glasses/day) were only associated with non-significant higher mortality rates of < 10% in the multivariable adjusted model (Table 2). Only 1.5% of the participants (3% of men and 0.6% of women) reported consuming more than 1,000 g per day (i.e., >5 glasses/day) of non-fermented milk. However, intakes above 1,000 g per day were associated with 34% (95% CI = 14, 59%) higher mortality rates compared to that in the group consuming < 200 g per day. More than 100 g of cheese per day compared to < 20 g per day was associated with 17% (95% CI = 5, 28%) lower mortality rates (Table 1). We found a sex-driven heterogeneity in the effects of cream (*p*-interaction = 0.002). Cream was associated with lower mortality among men (*p*-trend = 0.001), but not among women (*p*-trend = 0.23) (Supplementary Table 1). For women, a J-shaped association was observed with the lowest risk at 7 g per day. In contrast, among men, a lower mortality was observed up to 35 g per day of cream intake. At higher intakes, the risk of mortality leveled off (Supplementary Figure 1). Excluding individuals who potentially misreported their energy intakes and individuals reporting a substantial change in food habits before baseline (35% of the population) did not change observed associations (Supplementary Table 2).

**Table 2:** Association between dairy product intake and mortality.

		**Intake categories**							
		**1**	**2**	**3**	**4**	**5**	**6**	**7**	***P*-trend**
Non-fermented milk	Intake	0–200	200–400	400–600	600–800	800-−1,000	>1,000		
	*N*/deaths	11,655/2,853	8,011/2221	4,155/1,277	1,482/484	495/161	392/160		
	PY/deaths per 1,000 PY	22,0034/13.0	149,717/14.8	76,601/16.7	26,864/18.0	9,057/17.8	6,716/23.8		
	HR (basic model)	1.00	1.03 (0.97–1.09)	1.11 (1.04–1.19)	1.22 (1.10–1.34)	1.26 (1.07–1.48)	1.78 (1.52–2.09)		<0.001
	HR (full model)	1.00	1.00 (0.94–1.05)	1.05 (0.98–1.12)	1.08 (0.98–1.20)	1.09 (0.93–1.29)	1.34 (1.14–1.59)		0.002
	HR (energy-adjusted values)	1.00	0.99 (0.94–1.05)	1.07 (1.00–1.15)	1.12 (1.00–1.26)	1.18 (0.99–1.40)	1.34 (1.09–1.66)		<0.001
Fermented milk	Intake	0	0–100	100–200	200–300	>300			
	N/deaths	9,102/2,896	7,940/1,960	5,728/1,446	2,364/601	1,056/253			
	PY/deaths per 1,000 PY	166,162/17.4	149,226/13.1	108,745/13.3	44,894/13.4	19,962/12.7			
	HR (basic model)	1.00	0.88 (0.83–0.93)	0.82 (0.77–0.88)	0.82 (0.75–0.90)	0.79 (0.69–0.89)			<0.001
	HR (full model)	1.00	0.95 (0.89–1.00)	0.93 (0.87–0.99)	0.93 (0.85–1.02)	0.90 (0.79–1.03)			0.009
	HR (energy-adjusted values)	1.00	0.93 (0.88–0.99)	0.94 (0.88–1.00)	0.95 (0.87–1.04)	0.90 (0.79–1.03)			0.03
Cheese	Intake	0–20	20–40	40–60	60–80	80–100	>100		
	N/deaths	6,211/2,094	8,470/2,367	5,885/1,451	2,919/675	1,469/312	1,236/257		
	PY/deaths per 1,000 PY	112,758/18.6	157,667/15.0	111,316/13.0	55,428/12.2	28,243/11.0	23,578/10.9		
	HR (basic model)	1.00	0.88 (0.83–0.93)	0.83 (0.78–0.89)	0.85 (0.78–0.93)	0.79 (0.70–0.89)	0.82 (0.72–0.93)		<0.001
	HR (full model)	1.00	0.92 (0.86–0.97)	0.87 (0.81–0.94)	0.89 (0.81–0.97)	0.81 (0.72–0.92)	0.83 (0.72–0.95)		<0.001
	HR (energy-adjusted values)	1.00	0.94 (0.88–1.00)	0.90 (0.84–0.96)	0.90 (0.82–0.98)	0.83 (0.73–0.95)	0.82 (0.70–0.95)		<0.001
Cream	Intake	0–10	10–20	20–30	30–40	40–50	>50		
	N/deaths	12,483/3,421	6,929/1,785	3,405/931	1,624/451	831/268	918/300		
	PY/deaths per 1,000 PY	232,035/14.7	129,572/13.8	63,810/14.6	30,701/14.7	15,587/17.2	17,285/17.4		
	HR (basic model)	1.00	0.89 (0.84–0.94)	0.87 (0.81–0.94)	0.86 (0.78–0.94)	0.98 (0.86–1.11)	0.95 (0.84–1.07)		0.005
	HR (full model)	1.00	0.93 (0.88–0.98)	0.92 (0.85–0.99)	0.89 (0.80–0.98)	1.00 (0.88–1.14)	0.96 (0.85–1.08)		0.07
	HR (energy-adjusted values)	1.00	0.93 (0.88–0.99)	0.88 (0.81–0.96)	0.97 (0.87–1.08)	0.87 (0.74–1.01)	1.08 (0.94–1.24)		0.10
Butter	Intake	0	0–10	10–20	20–30	30–40	40–50	>50	
	N/deaths	14,826/4,034	3,671/858	2,185/555	1,780/508	1,349/418	768/220	1,611/563	
	PY/deaths per 1,000 PY	278,026/14.5	69,470/12.4	40,625/13.7	33,069/15.4	24,672/16.9	14,150/15.5	28,977/19.4	
	HR (basic model)	1.00	0.95 (0.88–1.02)	1.03 (0.94–1.13)	1.15 (1.05–1.26)	1.16 (1.05–1.29)	1.00 (0.87–1.14)	1.21 (1.11–1.33)	<0.001
	HR (full model)	1.00	0.98 (0.91–1.06)	1.00 (0.91–1.09)	1.08 (0.98–1.18)	1.05 (0.95–1.17)	0.90 (0.78–1.03)	1.04 (0.95–1.14)	0.46
	HR (energy-adjusted values)	1.00	0.96 (0.88–1.04)	1.00 (0.92–1.09)	1.02 (0.94–1.11)	1.12 (1.02–1.23)	0.98 (0.86–1.10)	0.99 (0.89–1.10)	0.39

*Basic model was adjusted for age, sex; Full model was adjusted for age, sex, method, season, energy, BMI, education, physical activity, smoking, alcohol habits, and diet (fruit and vegetables, meat, fiber, sugar-sweetened beverages). The individuals were categorized based on their energy-adjusted intake values (using the residual method) with standardized intakes at the median energy intake in this population (9.55 MJ). PY, person years of follow-up*.

The lactase persistence SNP rs4988235 was strongly associated with intake of non-fermented milk, but only weakly associated with intakes of the other dairy products (Table 3). CC genotype carriers (lactase non-persistence, i.e., lactose intolerance) had approximately 27% lower reported consumption of non-fermented milk than CT and TT carriers (222 vs. 279 and 283 g/day). In addition, CC genotype carriers had slightly higher intake of fiber, fruit and vegetables and lower BMI. CT/TT genotype carriers had a non-significant 8% higher mortality risk (HR = 1.08; 95% CI = 0.96, 1.23; *p* = 0.20) compared with that of CC genotype carriers (Table 3). The risk estimates for men and women were 1.16 (95% CI = 0.97, 1.38) and 1.01 (95% CI = 0.85, 1.20), respectively (p-interaction with sex = 0.28). Mendelian Randomization analysis using the lactase persistence SNP rs4988235 as instrumental variable showed that genetically determined milk intake was not statistically significantly associated with mortality in the whole cohort (OR per 100 g = 1.02; 95% CI = 0.96, 1.09; *p* = 0.48).

**Table 3:** Genetic variant (rs4988235) associated with lactase persistence and participant characteristics and mortality risk.

	**rs4988235 genotype**			
	**CC**	**TC**	**TT**	***P*-trend**
	**(lactase non-persistence)**	**(lactase persistence)**	**(lactase persistence)**	
*N*	1,038	7,459	13,737	
Age, years	57.6 (57.1–58.1)	57.9 (57.7–58.1)	58.1 (58.0–58.3)	0.003
Females	61.6%	62.5%	62.1%	0.77
BMI, kg/m^2^	25.2 (25.0–25.4)	25.4 (25.4–25.5)	25.6 (25.5–25.6)	<0.001
Energy intake, kcal/day	2,304 (2,270–2,339)	2,275 (2,262–2,288)	2,283 (2,273–2,293)	0.98
Carbohydrates, E%	45.3 (44.9–45.6)	45.1 (45.0–45.3)	44.9 (44.8–45.0)	0.005
Protein, E%	15.6 (15.4–15.7)	15.8 (15.7–15.8)	15.8 (15.7–15.8)	0.13
Fat, E%	39.1 (38.8–39.5)	39.1 (39.0–39.3)	39.3 (39.2–39.4)	0.03
Saturated fat, E%	16.9 (16.7–17.1)	16.9 (16.8–17.0)	17.0 (16.9–17.1)	0.04
Fiber, g/1,000 kcal	9.10 (8.94–9.25)	9.00 (8.94–9.06)	8.89 (8.85–8.93)	<0.001
Fruit and vegetables, g/day	378 (367–388)	371 (367–375)	366 (363–369)	0.003
Coffee, g/day	501 (478–524)	526 (518–535)	528 (521–534)	0.14
Meat, g/day	131 (128–135)	133 (131–134)	134 (133–135)	0.06
Fish, g/day	42.1 (40.2–44.0)	41.7 (41.0–42.5)	41.3 (40.8–41.9)	0.28
Non–fermented milk, g/day	222 (208–235)	279 (273–284)	283 (279–286)	<0.001
Fermented milk, g/day	83.3 (76.9–89.7)	86.6 (84.2–89.0)	88.4 (86.5–90.2)	0.08
Cheese, g/day	45.5 (43.8–47.2)	42.8 (42.1–43.4)	42.5 (42.0–42.9)	0.009
Cream, g/day	16.4 (15.4–17.3)	15.5 (15.1–15.9)	15.3 (15.0–15.6)	0.06
Butter, g/day	11.7 (10.4–12.9)	11.1 (10.6–11.5)	11.3 (11.0–11.6)	0.80
PY/deaths/deaths per 1,000 PY	19,658/268/13.6	139,195/2,079/14.9	257,023/3,814/14.8	
HR of mortality (95% CI): additive model	1.00	1.11 (0.97–1.26)	1.07 (0.95–1.22)	0.94
HR of mortality (95% CI): dominant model	1.00	1.08 (0.96–1.23)		0.20

*Mean and 95% CI for BMI were adjusted for age and sex; mean and 95% CI for dietary variables were adjusted for age, sex, energy, season, method. HR and 95% CI were adjusted for age and sex. PY, person years*.

## Discussion

In this middle-aged population from southern Sweden, we observed higher mortality rates mainly with very high intakes of non-fermented milk. Among the 1.5% of this population who consumed more than 1,000 g of non-fermented milk per day, we observed 34% higher mortality compared to that in the group consuming < 200 g per day. In contrast, fermented milk and cheese showed a protective association. Cream showed protective associations only among men, and butter was not associated with mortality.

The intake of dairy products has not consistently been associated with mortality (1–5). Due to differences in nutrient and fatty acid composition, processing, and fermentation among dairy products, it is crucial to examine them separately for health effects. Most studies have shown neutral or marginally beneficial associations, mainly with fermented dairy (5). There is substantial heterogeneity in the association between non-fermented milk consumption and mortality (1, 5). Most of the heterogeneity from systematic reviews is explained by a study of participants from the central part of Sweden where high intakes of non-fermented milk, similar to our study, were associated with higher mortality (9). In that study, consumption of three or more glasses of milk per day (i.e., 600 g/day) compared to less than one glass per day was associated with 93% higher mortality among women and 10% higher mortality among men (9). This contrasts with our results showing similar associations in both women and men. A cohort study from the northern part of Sweden, also revealed higher risk with high intakes of non-fermented milk (8). In that study, individuals consuming non-fermented milk 2.5 or more times per day had an 18% (95% CI: 6–33%) higher risk than those consuming milk less than once per week (8). The wide range of intakes with a high frequency of individuals consuming large amounts of dairy products in Sweden facilitates the possibility of finding significant associations between milk intake and mortality. The estimated mean intake of non-fermented milk was in our study 311 g/day among men and 256 g/day among women with baseline examination in 1991–1996. This is only slightly higher than the intake reported for the national food survey conducted in Sweden in 1997 (20), and only slightly higher than the mean intake of the cohort from the central part of Sweden, which was estimated using a food frequency questionnaire (FFQ) to be 290 g/day for men (examination in 1997) and 240 g/day for women (examination in 1987–90) (9). Increased mortality rates for non-fermented milk were also observed in the Nurse's Health Studies and Health Professional Follow-up Study with lower intakes compared to the studies conducted in Sweden (7).

With enough individuals consuming large amounts of milk in the MDC cohort, we could examine the mortality rates associated with very high intakes. Indeed, we observed a J-shaped association between non-fermented milk and mortality, but with a significant association observed only at very high intakes (above 1,000 g/day) of non-fermented milk.

Milk consumption was strongly related to other lifestyle factors in the MDC cohort, as well as in other Swedish cohorts, and the association was clearly attenuated after adjusting for these potential confounders; however, residual confounding could still be a problem. Since a similar confounding structure was observed in the other Swedish cohort, the potential harmful effects of very high intakes need to be examined in other populations or by conducting a large intervention study.

One way of overcoming the problem of confounding and reverse causation in observational studies is to use genetic markers as proxies for dietary intake. The rs4988235 genetic variant has been used as a proxy for intake of lactose-containing dairy products (21). Non-fermented milk contains much higher levels of lactose than fermented milk, and the cheese consumed in Sweden is generally free of lactose, whereas very small amounts are found in cream and butter. Lactose-intolerant individuals may be able to consume moderate amounts of lactose without symptoms, and we found 27% lower intake of non-fermented milk among participants with the lactase non-persistence CC genotype compared with a lactase persistence genotype. Only small differences between the genotype groups were observed for the other dairy products. Importantly, the food habits of participants with lactose intolerance differed with respect to not only lower intakes of dairy products but also somewhat higher intakes of fiber, fruit and vegetables. One explanation might be that individuals with lactase non-persistence must be more aware of what they eat. Therefore, this genetic variant is not only a marker of lower lactose intake but also potentially a marker of a healthier lifestyle, i.e., some pleiotropic effects occur, which needs to be considered when interpreting the results from studies in this field. In addition, a lower BMI was observed among individuals with the CC genotype. This has consistently been shown in other studies, providing strong support for the causal effect of higher lactose-containing dairy intake on increased BMI among adults (21). However, the association seems to be limited to increased lean body mass, not fat mass (22). We observed a non-significant 8% higher risk of mortality among participants with the TT/TC genotype compared to the lactase non-persistence CC genotype (i.e., lactose intolerance). The few studies that have examined the association between the genetic variant and mortality have found similar directions of the effects. In a study comprising 82,964 individuals of Danish descent with 7 years of follow-up, a risk estimate of 1.04 (95% CI: 0.93, 1.17) was observed for TT/CT vs. CC (23). In a cohort of 7,404 individuals born in Sweden with 14 years of follow-up, the risk estimate was 1.07 (95% CI: 0.80, 1.43) for TT/TC vs. CC (8). Although similar effect sizes were found in several cohort, a potential positive causal association between milk intake and mortality must be further explored.

The effects of dairy products are complex since the products differ in processing and nutrient contents. In the present study, fermented milk and cheese showed protective associations with mortality risk. In a meta-analysis of 11 observational studies, an inverse association was found between fermented dairy (mainly cheese) and mortality (5). However, this association was mainly driven by a Swedish study where women with high consumption of cheese (especially those consuming more than 20 grams per day) had a reduced mortality rate, while the associations in men were modest (9). In a study from the northern part of Sweden, cheese intake was also associated with lower mortality (8). A smaller Swedish study of 1,213 participants also showed a protective association with cheese (24). Cheese contains large amounts of fat, especially saturated fat, but the protective association with high intake of cheese may be explained by other compounds and nutrients found in hard cheese, including bioactive peptides, and reductions in the amounts of lactose and galactose by bacterial fermentation (25). In addition, the confounding structure in the Swedish population might contribute to the observed association. For example, cheese intake was strongly correlated with high educational status. The heterogeneity could also be because the composition of dairy products differs between countries. It could also be due to differences in diet assessment methods.

Studies examining the association between dairy intake and mortality have commonly used FFQs that differ in length (i.e., number of questions asked about different dairy foods) and the degree of detail (e.g., whether serving sizes were asked for) to estimate habitual dairy intake. Two limitations of the FFQ are that it heavily relies on memory and that standard serving sizes are often used. In the MDC cohort, a modified diet history method combining a 168 item FFQ with 7 day food records was used. For example, in the FFQ, questions were asked about milk used in coffee and tea and milk in porridge and breakfast cereals. In the food diaries, milk used in cooked foods and consumed as beverage was reported. This comprehensive and detailed data set on dairy food consumption is advantageous when examining the association between diet and disease risk. We previously found in the MDC cohort that high intakes of cheese and fermented milk were associated with reduced risk of cardiovascular diseases (15) and that high intakes of cream and high-fat fermented milk, and cheese among women, were associated with reduced risk of type 2 diabetes (26). Apart from the detailed diet assessment method, other major strengths of this study are that it included a large prospective cohort with a long follow-up, detailed data on potential confounders and complete mortality data.

Butter, which is a high-fat dairy product (~80% fat), was not associated with mortality in the present study. In the European EPIC cohort, high consumption of butter and margarine was related to higher mortality in individuals with diabetes (*n* = 6,384) but not in participants without diabetes (*n* = 258,911) (27). Butter intake was also associated with a modest higher risk in a cohort from the northern part of Sweden (8). In contrast, cream showed a protective association in men, which is not easily explained. To the best of our knowledge, no other studies have examined the association between cream intake and mortality. More studies examining these high-fat dairy products in relation to mortality risk are needed. In a meta-analysis of three available studies examining biomarkers of dairy fat measured in blood relation to mortality showed no clear association (28). In the study conducted in Sweden, a non-linear association was found with the lowest mortality rate observed around median concentration of serum pentadecanoic acid (15:0) (28).

In most nutritional recommendations worldwide, including those in Nordic countries, saturated fat intake is limited to 10% of energy intake (29). In the Swedish dietary guidelines, it is recommended to change from high-fat dairy to low-fat dairy (30). The effect of dairy foods on health is complex, and the food matrix seems to be important (25, 31). Dairy foods are a major source of energy and saturated fat, but also protein, vitamins and minerals in the diet (32). The results of the present study clarify the importance of dairy products for health and highlight the importance of distinguishing between different dairy foods when establishing recommendations. The findings do not support the current recommendation of limiting all high-fat dairy foods.

In conclusion, higher mortality rates were mainly observed among participants consuming more than 1,000 g of non-fermented milk per day. In contrast, high intakes of fermented milk and cheese were associated with lower mortality. Because dairy products differ in composition, it is important to examine them separately in relation to health and disease. The use of a genetic variant as an objective marker of lactose-containing milk intake should be examined in relation to mortality in a larger population.

## Data Availability Statement

The data analyzed in this study is subject to the following licenses/restrictions: The dataset for this article is not publicly available because of ethical and legal restrictions. Requests to access these datasets should be directed to Chair of the Steering Committee for the Malmö cohorts, see instructions at: https://www.malmo-kohorter.lu.se/english.

## Ethics Statement

The studies involving human participants were reviewed and approved by Ethics Committee at Lund University. The patients/participants provided their written informed consent to participate in this study.

## Author Contributions

ES devised the study, conducted the statistical analyses, and drafted the manuscript. EW and UE contributed important input to the analysis plan. YB performed the mendelian randomization analyses. All authors helped in interpretation of the results, gave final approval, and agree to be accountable for all aspects of work ensuring integrity and accuracy.

## Funding

This study was funded by the Albert Påhlsson Foundation.

## Conflict of Interest

The authors declare that the research was conducted in the absence of any commercial or financial relationships that could be construed as a potential conflict of interest.

## Publisher's Note

All claims expressed in this article are solely those of the authors and do not necessarily represent those of their affiliated organizations, or those of the publisher, the editors and the reviewers. Any product that may be evaluated in this article, or claim that may be made by its manufacturer, is not guaranteed or endorsed by the publisher.

## Abbreviations

BMI: body mass index
CI: confidence interval
FFQ: food frequency questionnaire
HR: hazard
*LCT*: lactase persistence gene
MDC: Malmö Diet and Cancer
PY: person-year
SNP: single nucleotide polymorphism
MET: metabolic equivalent of task.
