# Supplementary material for: Dairy Consumption, Lactase Persistence, and Mortality Risk in a Cohort From Southern Sweden
Source: Front Nutr. 2021 Nov 24;8:779034. doi: 10.3389/fnut.2021.779034 (PMC8652079; doi:10.3389/fnut.2021.779034)

**SUPPLEMENTARY MATERIALS**

**Supplemental table 1: Association between dairy product intake and mortality in men and women**

|  |  |  | **Intake categories** | | | | | | |  |
| --- | --- | --- | --- | --- | --- | --- | --- | --- | --- | --- |
|  |  |  | **1** | **2** | **3** | **4** | **5** | **6** | **7** | **P-trend** |
| **Men** | **Non-fermented milk** | **Intake** | 0-200 | 200-400 | 400-600 | 600-800 | 800-1000 | >1000 |  |  |
|  |  | **N/deaths** | 4140/1393 | 2837/1008 | 1664/619 | 732/275 | 278/109 | 296/128 |  |  |
|  |  | **Person-years** | 75544 | 51557 | 30086 | 12931 | 4942 | 5076 |  |  |
|  |  | **HR (95% CI)** | 1.00 | 0.98 (0.90-1.06) | 0.98 (0.89-1.08) | 1.00 (0.87-1.14) | 1.13 (0.92-1.38) | 1.28 (1.06-1.56) |  | 0.10 |
|  | **Fermented milk** | **Intake** | 0 | 0-100 | 100-200 | 200-300 | >300 |  |  |  |
|  |  | **N/deaths** | 4418/1726 | 2372/794 | 1826/600 | 870/275 | 461/137 |  |  |  |
|  |  | **Person-years** | 78615 | 43141 | 33705 | 16134 | 8540 |  |  |  |
|  |  | **HR (95% CI)** | 1.00 | 0.97 (0.89-1.05) | 0.91 (0.83-1.00) | 0.87 (0.77-0.99) | 0.88 (0.74-1.06) |  |  | 0.008 |
|  | **Cheese** | **Intake** | 0-20 | 20-40 | 40-60 | 60-80 | 80-100 | >100 |  |  |
|  |  | **N/deaths** | 2286/960 | 3063/1140 | 2228/738 | 1168/361 | 641/185 | 561/148 |  |  |
|  |  | **Person-years** | 39825 | 54765 | 40903 | 21830 | 12173 | 10640 |  |  |
|  |  | **HR (95% CI)** | 1.00 | 0.94 (0.86-1.02) | 0.90 (0.81-0.99) | 0.89 (0.79-1.01) | 0.86 (0.73-1.01) | 0.83 (0.69-1.00) |  | 0.007 |
|  | **Cream** | **Intake** | 0-10 | 10-20 | 20-30 | 30-40 | 40-50 | >50 |  |  |
|  |  | **N/deaths** | 4673/1701 | 2477/845 | 1279/467 | 673/213 | 391/144 | 454/162 |  |  |
|  |  | **Person-years** | 83719 | 45057 | 23273 | 12479 | 7178 | 8431 |  |  |
|  |  | **HR (95% CI)** | 1.00 | 0.90 (0.82-0.97) | 0.91 (0.82-1.01) | 0.77 (0.66-0.88) | 0.91 (0.76-1.08) | 0.84 (0.71-0.99) |  | 0.001 |
|  | **Butter** | **Intake** | 0 | 0-10 | 10-20 | 20-30 | 30-40 | 40-50 | >50 |  |
|  |  | **N/deaths** | 5604/1949 | 1049/346 | 732/233 | 611/239 | 557/215 | 378/134 | 1016/416 |  |
|  |  | **Person-years** | 102199 | 19246 | 13288 | 10856 | 9866 | 6847 | 17834 |  |
|  |  | **HR (95% CI)** | 1.00 | 0.98 (0.88-1.10) | 0.98 (0.86-1.13) | 1.18 (1.03-1.36) | 1.03 (0.90-1.19) | 0.92 (0.77-1.10) | 1.06 (0.94-1.18) | 0.33 |
| **Women** | **Non-fermented milk** | **Intake** | 0-200 | 200-400 | 400-600 | 600-800 | 800-1000 | >1000 |  |  |
|  |  | **N/deaths** | 7515/1460 | 5174/1213 | 2491/658 | 750/209 | 217/52 | 96/32 |  |  |
|  |  | **Person-years** | 144496 | 98164 | 46518 | 13934 | 4115 | 1640 |  |  |
|  |  | **HR (95% CI)** | 1.00 | 1.01 (0.93-1.09) | 1.11 (1.00-1.22) | 1.19 (1.02-1.38) | 1.00 (0.76-1.33) | 1.48 (1.04-2.11) |  | 0.007 |
|  | **Fermented milk** | **Intake** | 0 | 0-100 | 100-200 | 200-300 | >300 |  |  |  |
|  |  | **N/deaths** | 4684/1170 | 5568/1166 | 3902/846 | 1494/326 | 595/116 |  |  |  |
|  |  | **Person-years** | 87552 | 106089 | 75043 | 28761 | 11423 |  |  |  |
|  |  | **HR (95% CI)** | 1.00 | 0.94 (0.87-1.02) | 0.95 (0.86-1.04) | 0.99 (0.87-1.12) | 0.93 (0.77-1.13) |  |  | 0.42 |
|  | **Cheese** | **Intake** | 0-20 | 20-40 | 40-60 | 60-80 | 80-100 | >100 |  |  |
|  |  | **N/deaths** | 3925/1134 | 5407/1227 | 3657/713 | 1751/314 | 828/127 | 675/109 |  |  |
|  |  | **Person-years** | 72937 | 102906 | 70416 | 33599 | 16070 | 12939 |  |  |
|  |  | **HR (95% CI)** | 1.00 | 0.89 (0.82-0.97) | 0.84 (0.76-0.93) | 0.88 (0.77-1.01) | 0.76 (0.63-0.92) | 0.84 (0.68-1.04) |  | 0.001 |
|  | **Cream** | **Intake** | 0-10 | 10-20 | 20-30 | 30-40 | 40-50 | >50 |  |  |
|  |  | **N/deaths** | 7810/1720 | 4452/940 | 2126/464 | 951/238 | 440/124 | 464/138 |  |  |
|  |  | **Person-years** | 148322 | 84519 | 40539 | 18222 | 8410 | 8855 |  |  |
|  |  | **HR (95% CI)** | 1.00 | 0.96 (0.89-1.05) | 0.92 (0.83-1.02) | 1.03 (0.90-1.19) | 1.13 (0.94-1.36) | 1.16 (0.97-1.38) |  | 0.23 |
|  | **Butter** | **Intake** | 0 | 0-10 | 10-20 | 20-30 | 30-40 | 40-50 | >50 |  |
|  |  | **N/deaths** | 9222/2085 | 2622/512 | 1453/322 | 1169/269 | 792/203 | 390/86 | 595/147 |  |
|  |  | **Person-years** | 175835 | 50226 | 27338 | 22214 | 14807 | 7304 | 11144 |  |
|  |  | **HR (95% CI)** | 1.00 | 0.98 (0.89-1.08) | 1.01 (0.89-1.13) | 1.00 (0.88-1.13) | 1.08 (0.93-1.25) | 0.86 (0.69-1.07) | 1.00 (0.84-1.20) | 0.92 |

Adjusted for age, sex, method, season, energy, BMI, education, physical activity, smoking, alcohol habits, dietary intakes (fruit and vegetables, meat, fiber, and sugar-sweetened beverages)

**Supplemental table 2: Association between dairy product intake and mortality after excluding potential misreporters of energy and past food habit changers**

|  | **Intake categories** | | | | | |  |  |  |
| --- | --- | --- | --- | --- | --- | --- | --- | --- | --- |
|  | **1** | **2** | **3** | **4** | **5** | **6** | | **7** | **p-trend** |
| **Non-fermented milk** | 1.00 | 1.03 (0.96-1.11) | 1.07 (0.98-1.16) | 1.06 (0.94-1.21) | 1.13 (0.92-1.38) | 1.38 (1.12-1.71) | |  | 0.007 |
| **Fermented milk** | 1.00 | 0.94 (0.87-1.01) | 0.93 (0.86-1.01) | 0.95 (0.85-1.06) | 0.90 (0.76-1.06) |  | |  | 0.07 |
| **Cheese** | 1.00 | 0.91 (0.85-0.98) | 0.87 (0.80-0.95) | 0.85 (0.76-0.95) | 0.79 (0.68-0.92) | 0.84 (0.70-1.00) | |  | <0.001 |
| **Cream** | 1.00 | 0.92 (0.86-0.99) | 0.91 (0.83-0.99) | 0.88 (0.78-0.99) | 0.96 (0.83-1.12) | 0.88 (0.76-1.02) | |  | 0.01 |
| **Butter** | 1.00 | 0.96 (0.88-1.06) | 1.01 (0.90-1.12) | 1.10 (0.99-1.23) | 1.10 (0.97-1.23) | 0.90 (0.76-1.05) | | 1.02 (0.91-1.14) | 0.46 |

Adjusted for sex, age, method, season, energy, BMI, education, physical activity, smoking, alcohol habits, intake of fruit and vegetables, meat, fiber, sugar-sweetened beverages

**Supplemental figure 1: Association between intakes (grams per day) of fermented milk (A), cheese (B), cream (C), butter (D) and mortality rate modelled using a restricted cubic spline in 26190 participants from the Malmö Diet and Cancer Study.** The model was adjusted for adjusted for age, sex, method, season, energy, BMI, education, physical activity, smoking, alcohol habits, and dietary intakes (fruit and vegetables, meat, fiber, sugar-sweetened beverages). Solid line is Hazard Ratio and dotted line is 95% CI.


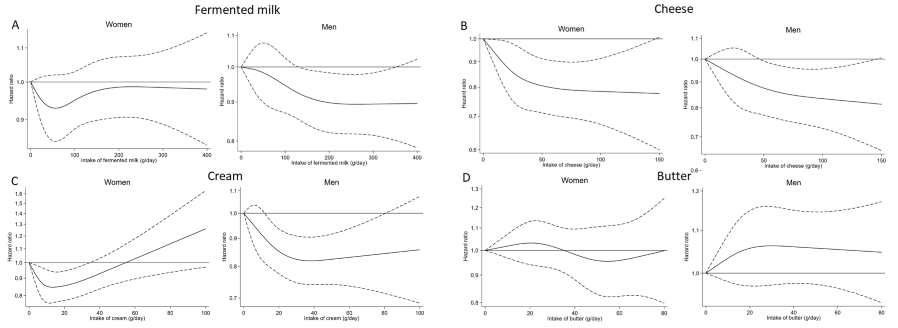

Supplement: Supplementary file 1 [file Data_Sheet_1.docx]
